# Impacts of Mobility Dogs on Kinematics during Ambulation: A Quantitative Study

**DOI:** 10.3390/vetsci8110250

**Published:** 2021-10-26

**Authors:** Kayla Altman, Samantha Glumm, Kendall Stainton, Ellen Herlache-Pretzer, Stacey Webster, Melissa Y. Winkle

**Affiliations:** 1 Master of Science in Occupational Therapy Program, College of Health and Human Services, Saginaw Valley State University, University Center, MI 48710, USA; knaltman@svsu.edu (K.A.); sjshafer@svsu.edu (S.G.); kastaint@svsu.edu (K.S.); srwebste@svsu.edu (S.W.); 2 Dogwood Therapy Services, Albuquerque, NM 87120, USA; melissa@dogwoodtherapy.com

**Keywords:** mobility dog, kinematics, assistive device, kinovea, ambulation

## Abstract

While prior research has explored various physiological consequences associated with assistive device use for ambulation, limited research has specifically explored the impact of mobility dog partnership on human kinematics. This descriptive study examined the impact of mobility dog partnership on kinematics of individuals in the normal young adult population. Sixteen participants were video recorded while walking in a straight line for 3.7 m (12 feet) under three different conditions (ambulating with no device, ambulating with a standard cane on the left side, and ambulating with a mobility dog on the left side). Differences between joint angles under each of the conditions were analyzed. Statistically significant differences were found in left elbow flexion when comparing ambulating with a cane versus ambulating with no device; left shoulder abduction when comparing ambulating with a cane versus ambulating with a mobility dog, ambulating with a mobility dog versus no device, and ambulating with a cane versus no device; and left hip extension when comparing ambulating with a mobility dog versus no device, and when ambulating with a mobility dog versus a cane. These findings suggest that providers should evaluate and monitor potential negative impacts of assistive devices such as mobility dogs on human kinematics.

## 1. Introduction

According to a report published by the World Health Organization in 2018 [1], 15% of the world’s population lives with a disability. Mobility-related disabilities are the most prevalent, with 13.7% of Americans experiencing some form of mobility impairment [2]. Mobility devices (including canes, walkers, and wheelchairs) are often used by individuals experiencing mobility disabilities [3] to enhance quality of life and improve functional status by improving balance and mobility, decreasing weight bearing, alleviating pain, or compensating for weakness or impaired motor control of the legs [4]. However, providers recommending mobility devices must be aware of the demands that mobility device use places on the cognitive, neuromotor, and neuromuscular systems. Additionally, proper fitting of an assistive device to the user is important [5], as any restriction, imbalance, or misalignment that occurs when an individual uses any type of assistive device can have an adverse effect on the efficiency and safety of device use [6,7]. Specifically, improper use of, and education regarding, mobility aids such as canes and walkers can lead to negative outcomes including increased fall risk, injury due to improper fit of selected device, and poor posture during usage [5,7,8]. Yet with careful selection, fitting, and training, mobility devices can have a positive impact on kinematics during ambulation among persons with mobility impairments. For example, Krautwurst et al. used video recordings to evaluate the joint angles of the lower extremities of individuals with bilateral cerebral palsy using mobility devices for ambulation. Results indicated that the type of device utilized had a significant impact on multiple joints under evaluation, with some joints displaying improved kinematics as a result of device use [9].

While mobility devices including canes, walkers, and wheelchairs are commonly used to provide assistance with functional mobility, some individuals with mobility disabilities may choose to partner with a specific type of assistance dog called a mobility dog. Mobility dogs can assist wheelchair users with propulsion assistance. They can also assist ambulatory individuals with their balance and gait while walking by acting as a brace or counterbalance for individuals with limitations in balance and strength. Mobility dogs performing this type of work typically wear a special harness that allows them to provide balance assistance in a manner that is safe for both the handler and the dog [10]. For the purposes of this study, the term “mobility dog” will be used to specifically refer to dogs that provide bracing and mobility support to people who are ambulatory.

Prior research has explored the impacts of mobility dog partnership on various aspects of mobility of the human partner, including speed, joint loading, and muscular demand. Wheelchair users partnered with a mobility dog trained to assist them in ascending ramps have demonstrated decreases in mechanical load and muscular demands of the upper extremity [11,12]. Blanchett et al. found that, among persons with various physical disabilities who were ambulatory, mobility dog partnership resulted in improved performance on the 10-m walk test, the timed up-and-go (TUG) test, and the stair ascent test [13]. In a case study by Abbud et al. involving two individuals with ataxia, mobility dog partnership was shown to improve walking patterns and promote safer, more independent mobility [14]. Research by Rondeau et al. examining the results of an intervention involving a trained “rehabilitation dog” concluded that all four study participants (stroke survivors) demonstrated improved walking speed and gait patterns when ambulating with the rehabilitation dog versus with a cane [15]. Noguchi et al. explored the kinematics of sit to stand transfers by persons with rheumatoid arthritis. Specifically, participants completed transfers without assistance, transfers with a cane, and transfers with a mobility dog. The authors concluded that mobility dog partnership leads to improved transfer performance for many individuals with functional disabilities and physical impairments [16].

However, research has also indicated that mobility dog partnership can have potentially harmful impacts on the kinematics of their human partners. For example, in their study examining the effects of mobility dog partnership on the kinematics of individuals using wheelchairs, Coppinger et al. found that completion of mobility dog tasks (specifically, pulling a wheelchair and opening a door for a person in a wheelchair) placed significant physical strain on both the mobility dog and the wheelchair user [17]. More recently, Kalish and colleagues demonstrated an increase in joint pain, particularly in the shoulder, among blind individuals after switching from a long cane to a guide dog for mobility [18,19]. While the team did not explicitly state that the pain was due to guide dog partnership, the researchers suggested that this finding may indicate the need for more ergonomic harness designs and ergonomics education for guide dog users [18].

Together these findings suggest that, similar to other types of mobility devices, mobility dog partnership has the ability to impact the human partner positively, or negatively, during ambulation. As mobility with or without devices involves sustained posturing and positioning of joints, it is important to understand how use of mobility aids or mobility dog partnerships impact joint angles. Therefore, the purpose of this descriptive study was to examine the impact of mobility dog partnership on kinematics of individuals within the normal adult population. Specifically, this study examined the differences, if any, between individuals’ joint angles when walking with no mobility device, versus walking with a standard cane, versus walking with a trained mobility dog wearing a mobility harness. It was hypothesized that there would be a statistically significant difference between mean joint angle measurements when comparing the joint angles under each of the three conditions for each joint measured. Separate comparisons were completed for each joint on both the left side and the right side.

## 2. Materials and Methods

### 2.1. Participant Recruitment

Participants were recruited by word of mouth and flyers posted at the researchers’ university. Participants had to be between the ages of 18–30 years old and enrolled at the researchers’ university at the time of the study. This age range was selected in order to improve consistency of the data collection results, as participants who were closer in age were more likely to have physical similarities. In addition, participants must have been able to grasp an assistive device handle with their left hand when ambulating and be able to walk without assistance from a mobility device such as a walker, cane, or brace for a distance of 3.7 m (12 feet). People with a history of a condition impacting range of motion in the shoulder, elbow, wrist, hip, knee, and/or ankle joints were excluded from the study. The authors aimed to recruit a total of 15–20 participants. This decision was based off three previously conducted gait analysis studies which utilized 16, 20 and 20 participants respectively [20,21,22].

Written informed consent was obtained from all participants prior to the start of the study. The study was approved in advance by the Saginaw Valley State University Institutional Review Board (approval number 1450595) and the Saginaw Valley State University Institutional Animal Care and Use Committee (approval number 1480954). Seventeen people (15 women and 2 men) were recruited for the study; a total of 16 participants completed all portions of the study. One person (male) who enrolled did not complete all portions of data collection and was thus excluded from the final results. The mean age of the study participants was 22.5 + 1.83 (SD) years old, with their mean height being 163 + 9.50 (SD) centimeters.

### 2.2. Data Collection

Data was collected in a private room at the researchers’ university. The room was approximately 9 m wide and 12 m long, and contained a hard, flat, even walking surface with no obstacles, and adequate lighting. Tape marks were placed along the walking path (a straight line) at the 0.9, 1.8, 2.7, and 3.7 m (3, 6, 9, and 12 feet) marks to provide consistent, equidistant points of reference for obtaining multiple joint angle measurements to establish a mean measurement for each joint. The distance of 3.7 m was selected for the walking distance based on recommendations by Pirker and Katzenschlager stating that clinical examination of gait should involve observation while ambulating at least several meters without obstacles [23]. Furthermore, this distance is comparable to that of the distance an individual is required to walk to complete many common gait assessments such as the TUG (3 m) [24], Functional Ambulation Categories (10 feet) [25], Tinetti Balance Test (15 feet) [26], and Functional Gait Index (20 feet) [27]. A four-year-old male golden retriever certified as an assistance demonstration dog for one of the researchers (EHP) through an Assistance Dogs International-accredited assistance dog training organization was utilized for the mobility dog portion in this study. Consent was obtained from the dog’s trainer/owner (EHP) for the dog’s inclusion in this study. The dog had previous experience working with people with a variety of disabilities and was trained in mobility dog work. The mobility dog was fitted with an adjustable Bold Lead Designs mobility support harness with a rigid handle. An adjustable standard straight cane was utilized for the cane walking condition.

**Figure 1:**
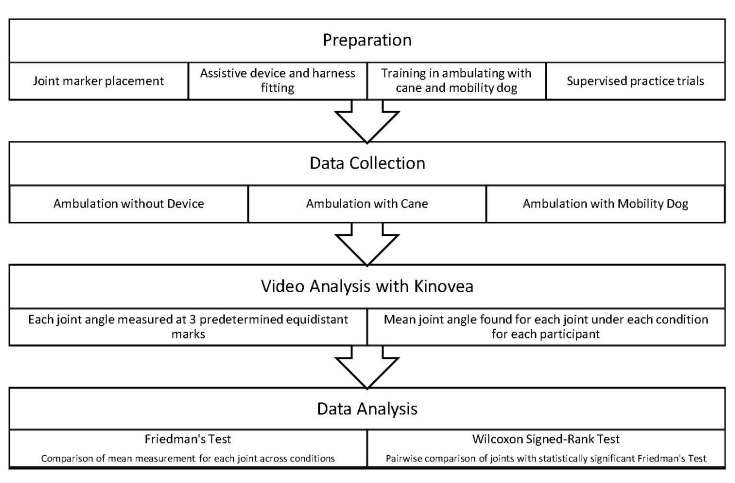
Diagram of study design.

Participation in the study involved a single session that lasted approximately 15–20 min (refer to Figure 1 for diagram illustrating study design). First, each participant completed a questionnaire gathering information regarding biological sex, age, and height. Participants then had stickers placed on the T7 spinal joint, bilateral glenohumeral joints, medial epicondyle of humerus bilaterally, bilateral lateral aspects of the iliac crests, bilateral lateral and medial epicondyles of the femurs, and bilateral lateral and medial malleoli of the ankles. Participants were then fitted for the standard cane and mobility harness according to standard fit guidelines [28,29]. Specifically, for both the cane and harness conditions, the device was fitted so that the top of the device lined up with the wrist crease, and the elbow was positioned in slight flexion when holding the device. When ambulating with the mobility dog, the dog was positioned with the dog’s shoulders slightly in front of the participants’ hips to allow for comfortable positioning of the elbow and shoulder. Participants received verbal and visual instructions regarding proper ambulation while utilizing the cane and mobility support harness. Because the mobility dog included in the study was trained to walk on a handler’s left side, it was decided that all participants would be required to complete ambulation with the dog on their left side as well as with the cane in their left hand. Participants completed supervised practice trials with the cane and the mobility dog prior to filming.

**Figure 2:**
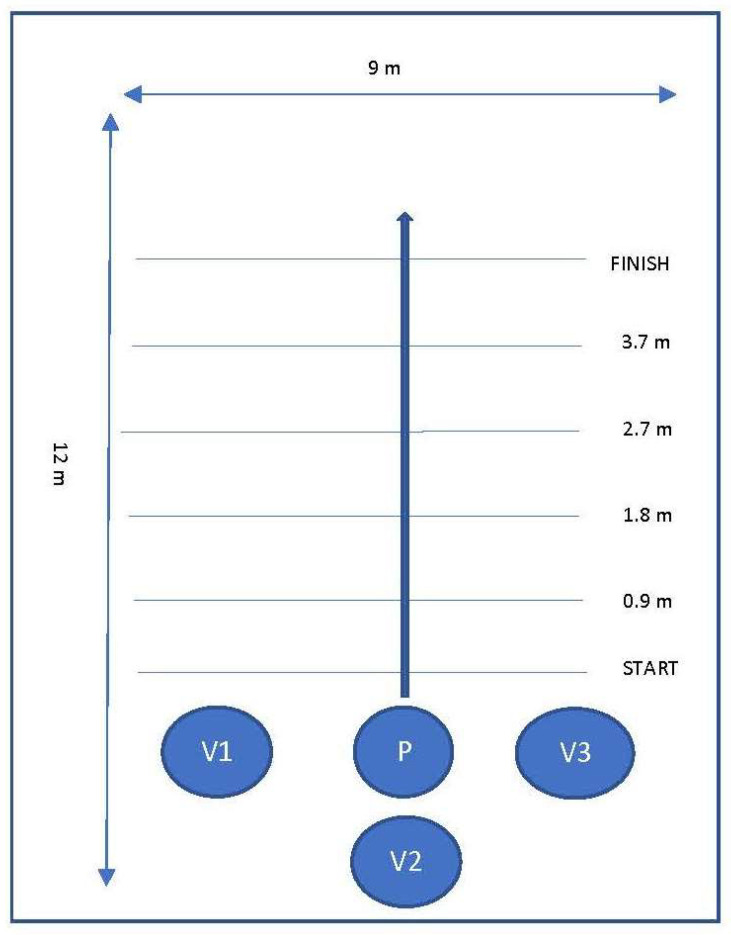
Diagram of layout used for data collection under each condition. Participant (P) was positioned behind the start line, to ensure they were in full stride by the time they hit the first joint angle measurement line (0.9 m). Participants were instructed to continue walking until they passed the finish line, to ensure that their kinematics were not impacted artificially by coming to a stop before the final joint measurement line (3.7 m). Researchers with iPhones (V1, V2, V3) were positioned to the left, right, and immediately behind the participant, and moved at the same speed as the participant throughout each condition.

The researchers’ iPhones were used to simultaneously video participants from the right, left, and back sides while they ambulated in each condition. For the first condition, the participants ambulated with no device for a distance of 3.7 m (12 feet). This process was then repeated, with the participants ambulating the same distance, utilizing the standard cane on their left side. Finally, the above process was repeated for the third condition, while the participants ambulated with the mobility dog on their left side (refer to Figure 2 for diagram of the layout used for data collection under each condition).

### 2.3. Data Analysis

Kinovea, an open-source software program created by Joan Charmant, allows for measurement, evaluation, and analysis of passive and active range of motion of various joint angles captured on video. Kinovea v.0.8.1 was used for video analysis in the present study [30]. Kinovea has been shown to have high validity, interrater reliability, and intra-rater reliability for analysis of joint angles in both upper and lower extremities [31,32,33]. Kinovea was used to determine the degrees of range of motion for the following joint angles in each condition: bilateral elbow flexion; left shoulder abduction; thoracic spinal curvature; right hip flexion; left hip extension; bilateral knee flexion; and bilateral ankle plantarflexion. Each joint angle measurement was taken at the time of the first right heel strike on or after the marked distances of 0.9, 1.8, 2.7, and 3.7 m (3, 6, 9, and 12 feet) from the start point. Each participants’ joint measurements at each of these distances were then averaged under each condition (ambulating without a mobility device, with a standard cane, and with a mobility dog).

For each joint, Friedman’s test was used to compare these average measurements for all participants across all conditions to determine if statistically significant differences were present. Separate analyses were completed for the left side and right side. An alpha level of 0.05 was used to determine significance. For those comparisons in which a statistically significant difference was identified, post hoc testing was completed using a two-tailed Wilcoxon Signed-Ranks test with a Bonferroni-corrected alpha level of 0.0167 to determine the conditions between which there were statistically significant differences.

## 3. Results

### 3.1. Significant Differences in Joint Position across Conditions

Separate comparisons were completed for each joint on both the left side and the right side. Descriptive statistics and Friedman’s tests were completed for each side for each joint under each condition. Refer to Table 1 and Table 2 for a summary of mean and standard deviations of joint measurements, and p-values obtained from Friedman’s tests, across participants’ joint angles under all conditions. An alpha level of 0.05 was utilized for significance testing. Statistically significant differences were found for left elbow flexion, left shoulder abduction, and left hip extension (*p* = 0.00, *p* = 0.00, *p* = 0.03). Thus, the researchers rejected the null hypotheses for these three differences.

### 3.2. Significant Differences in Joint Positions within Conditions

Post hoc testing with the Wilcoxon Signed-Rank test was utilized to determine the specific conditions under which statistically significant differences in joint angles existed. Refer to Table 3 for results of post-hoc testing. Following the guidelines provided by Kim [34], a Bonferroni-corrected alpha level was utilized. After dividing the previously-selected alpha level (0.05) by the number of comparisons (3), an alpha level of 0.0167 was utilized for significance testing. Statistically significant differences were found in several joint positions and conditions on the left side, leading to rejection of the corresponding null hypotheses. Specifically, there were statistically significant differences in left elbow flexion when comparing ambulating with a cane versus ambulating with no device (*p* = 0.001). There were also statistically significant differences in left shoulder abduction when comparing ambulating with a mobility dog versus ambulating with a cane, ambulating with a mobility dog versus no device, and ambulating with a cane versus no device (*p* = 0.001, *p* = 0.000, and *p* = 0.002). Finally, there were also statistically significant differences in left hip extension when comparing ambulating with a mobility dog versus a cane, as well as when ambulating with a mobility dog versus no device (*p* = 0.016, *p* = 0.006). The researchers rejected the null hypotheses that corresponded to these six joint angles and conditions. However, they failed to reject the remaining null hypotheses that were related to all other joint angles when compared under other conditions.

## 4. Discussion

The results of the present study suggest that ambulating with an assistive device, including a mobility dog, may impact kinematics (i.e., joint angle measurements) of select joints. Statistically significant differences were found in left shoulder abduction joint angles across all conditions, in left elbow flexion joint angles when comparing the use of a cane to no device during ambulation, and in left hip extension when comparing ambulating with a dog to no device and to a cane.

The statistically significant difference found in left elbow flexion when comparing ambulating with a cane to no device could imply that left elbow flexion is impacted due to the nature of ambulating with a cane. When walking with a cane, the individual must lift the cane off the ground and place it ahead of them, leading to increased elbow flexion when lifting the cane while ambulating, when compared to ambulating with no device. The results of the testing indicated there was no statistical significance in left elbow flexion when ambulating with no device versus with the mobility dog.

It is also logical that there was no statistically significant difference found when comparing measurements obtained when comparing walking with no device versus walking with a mobility dog. When walking with a mobility dog in heel position, the individual does not move or lift the arm to reposition the device when moving through the environment; rather, the arm stays in constant contact with a stable “surface” (i.e., harness handle) as the dog moves through the environment. This is consistent with prior research suggesting that mobility dogs require fewer movements for use when compared to traditional mobility devices such as canes, which may make them preferable for people with disabilities that impact their ability to produce coordinated, controlled movements [14,16].

It appears that left shoulder abduction was impacted by both cane use and mobility dog use, as the users may have had to position the arm in slight abduction to accommodate for the added width of the device (cane/dog with harness). These findings regarding the potential impact of ambulation with a mobility dog on the shoulder makes sense in context with the results of prior research focused specifically on guide dog users, which highlighted negative musculoskeletal effects (most notably at the shoulder) associated with the use of standard guide harnesses [18,19]. However, it is important to note that the position of the upper extremity (particularly at the shoulder) during harness use differs for guide dog handlers versus mobility dog handlers whose dogs are trained specifically for balance assist. Because a guide dog is trained to lead out, the handler’s upper extremity is positioned in shoulder flexion and slight abduction (which can vary depending upon the design of the harness handle (i.e., offset vs straight harness handle)). Additionally, traditional guide dog harnesses are not height-adjustable [35]. For the mobility harness used in the present study, the mobility dog remained in heel position (i.e., shoulders at or only slightly ahead of handler’s hip) throughout filming, resulting in minimal shoulder flexion. This ensures that during mobility dog work, any forces transmitted through the harness are properly transmitted throughout the dog’s core. Additionally, the harness handle was able to be adjusted based upon the handler’s height to allow for minimal elbow flexion [29]. These results suggest that mobility harness designs could affect upper extremity kinematics and have long-term musculoskeletal impacts, particularly at the shoulder. Therefore, it may be valuable to have professionals skilled in human kinematics and biomechanics included in decisions regarding harness design and fit for humans working with mobility dog partners.

Interestingly, in the present study, significantly decreased hip extension was found when participants ambulated with the dog versus ambulating with no device or with a cane. It is possible that this is due to the modulating effect of the dog’s speed on the participants’ pace and stride length (and therefore joint angle). This is in contrast to prior findings supporting the positive impacts of mobility dog partnership on walking speed and gait patterns in persons with disabilities [13,15]. In the present study, the dog’s trainer/owner walked with the dog to ensure dog and participant safety and proper positioning. However, this could have artificially regulated the dog’s speed throughout testing, which may have which may have differed from participants’ typical pace and stride length. Future research should continue to explore how stride length and joint angle is impacted in established mobility dog partnerships.

Limitations to the study include the mobility dog’s trainer/owner walking with the participants and the mobility dog during testing. In addition to affecting pace and stride length, it is possible that, in the absence of this trainer/owner, a mobility dog-human team may not consistently maintain proper positioning when working in real life, which could result in different outcomes being observed. Another limitation to this study is the participants’ inexperience ambulating with mobility devices. The participants could have potentially been focusing more on utilizing the mobility devices correctly when ambulating, instead of walking with their typical movement patterns (Hawthorne effect [36]). Thus, it is possible that individuals with more experience of ambulating with mobility devices or mobility dogs may have results that are more consistent with their typical movement patterns, which could cause different outcomes to be observed.

Future research should continue to explore the impact of mobility dogs on human kinematics during ambulation. Specifically, future research should include participants with disabilities who are partnered with mobility dogs and experienced with the use of mobility aids/devices. This will allow a better understanding of how mobility dog partnership may impact the kinematics of individuals with disabilities. Additionally, a longitudinal study would provide information regarding if and how these kinematics may change over time. This knowledge could be used to develop guidelines for dog/harness fit and handler training and education to reduce potential negative impacts on human user’s kinematics. Additionally, future research should be carried out involving different types of mobility harnesses, to explore if different styles of harnesses impact human kinematics differently and if so, how they impact them differently.

## 5. Conclusions

The results of this study are consistent with prior research concluding that mobility device use can impact human kinematics in select joints during ambulation. Specifically, the findings suggest that mobility dog partnership may differentially impact kinematics as compared to cane use or no device use. Based upon these findings, it is suggested that individuals involved in the assessment, training, and placement of human-mobility dog teams carefully monitor human kinematics, particularly within the shoulder and hip of the side on which the dog works, to ensure that partnership does not have negative impacts on the human’s biomechanics. The use of carefully-fitted harnesses customized to the human user’s height may also decrease the potential for long-term negative impacts of mobility dog partnership on human kinematics. Furthermore, it may be beneficial to involve professionals with advanced knowledge and training in human kinematics throughout the process, to decrease the risk of short- and long-term injuries due to poor harness fit and use. However, because this study involved healthy young adults, it is important that this study be replicated with a population of individuals with disabilities.

## Figures and Tables

**Table 1:** Mean and standard deviation of specified joint angles for all participants under each condition, measured in degrees, and calculated *p*-values obtained from Friedman’s test, for right side.

Joint Angle	No Device	Cane	Dog	*p*-Value
Elbow Flexion, Right	12.80 (3.85)	13.75 (6.71)	12.11 (4.75)	0.38
Shoulder Abduction, Right	-- ^1^	-- ^1^	-- ^1^	
Thoracic Spinal Curvature, Right	1.29 (0.86) ^2^	1.29 (0.97) ^2^	1.56 (1.04) ^2^	0.34
Hip Flexion, Right	16.40 (3.55)	15.40 (5.68)	16.88 (4.69)	0.74
Hip Extension, Right	-- ^3^	-- ^3^	-- ^3^	
Knee Flexion, Right	10 (3.28)	9.29 (3.19)	10.58 (4.84)	0.83
Ankle Plantarflexion, Right	15.4 (6.11)	16.10 (6.75)	14.40 (7.67)	0.21

^1^ Measurements not completed due to positioning of right arm in neutral position at side of body during trial; ^2^ Indicates direction of spinal curvature; ^3^ Measurements not completed due to positioning of hip joint at time of measurement (right heel strike).

**Table 2:** Mean and standard deviation of specified joint angles for all participants under each condition, measured in degrees, and calculated *p*-values obtained from Friedman’s test, for left side.

Joint Angle	No Device	Cane	Dog	*p*-Value
Elbow Flexion, Left	18.79 (11.54)	36.77 (12.88)	28.73(12.38)	0.00 ^3^
Shoulder Abduction, Left	9.58 (3.60)	12.88 (3.81)	20.54 (4.75)	0.00 ^3^
Thoracic Spinal Curvature, Left	-- ^1^	-- ^1^	--^1^	
Hip Flexion, Left	-- ^2^	-- ^2^	-- ^2^	
Hip Extension, Left	14.54 (5.66)	15.42 (5.60)	10.42 (4.30)	0.03 ^3^
Knee Flexion, Left	19.31 (9.01)	15.71 (7.53)	20.10 (6.36)	0.44
Ankle Plantarflexion, Left	12.09 (7.22)	9.44 (5.64)	13.10 (5.54)	0.17

^1^ No thoracic spinal curvature to left side present. ^2^ Measurements not completed due to positioning of hip joint at time of measurement (right heel strike). ^3^ Indicates the presence of a statistically significant difference (*p* < 0.05) in the joint between the three conditions on the indicated side.

**Table 3:** Results of post-hoc Wilcoxon Signed-Rank Test for joint angles with statistically significant differences (as identified through Friedman’s Test).

Condition	Elbow Flexion (L)	Shoulder Abduction (L)	Hip Extension (L)
Dog-Cane	0.023	0.001 ^1^	0.016 ^1^
Dog-No Device	0.044	0.000 ^1^	0.006 ^1^
Cane-No Device	0.001 ^1^	0.002 ^1^	0.586

^1^ Indicates the presence of a statistically significant difference (*p* < 0.0167) in the joint between the two conditions on the indicated side.

## Data Availability

Data available on request due to participant privacy restrictions.
